# Lasting Impacts: Pre- and Postnatal PBDE Exposures Linked to IQ Deficits

**DOI:** 10.1289/ehp.121-a58

**Published:** 2013-02-01

**Authors:** Kellyn S. Betts

**Affiliations:** Kellyn S. Betts writes about environmental contaminants, hazards, and technology for solving environmental problems for publications including *EHP* and *Environmental Science & Technology*.

The largest study to investigate the effects of human exposure to polybrominated diphenyl ether (PBDE) flame retardants reports evidence of impaired neurobehavioral development in connection to both prenatal and childhood exposures [*EHP* 121(2):257–262; Eskenazi et al.]. Associations were greatest for attention, fine motor coordination, and cognition in school-age children.

The study focused on chemicals associated with pentaBDE, a mixture of PBDEs that was used in polyurethane foam padding in furniture and infant products manufactured before 2005. These flame retardants can be released into home environments throughout products’ life spans, and children are disproportionately likely to be exposed to them through hand-to-mouth activity. PBDEs are chemically similar to polychlorinated biphenyls (PCBs), which are associated with similar neurodevelopmental impairments.

**Figure:**
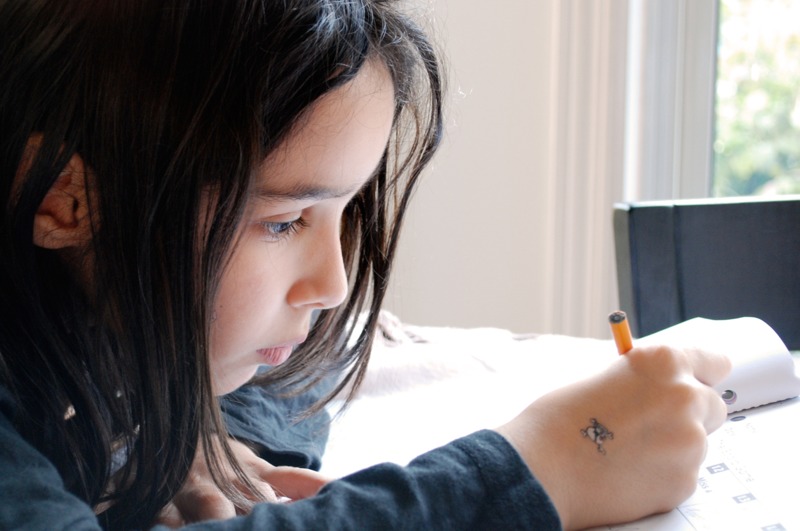
Childhood exposure to PBDEs may adversely affect IQ independently of prenatal exposure. © Claudia L. Kleinman

The new study included mothers and children participating in the ongoing CHAMACOS (Center for the Health Assessment of Mothers and Children of Salinas) cohort study, which in 1999 and 2000 recruited more than 600 pregnant women living in California’s agricultural Salinas Valley. Most of the mothers were Mexican immigrants, and neurobehavioral assessments were conducted by bilingual psychometricians in the dominant language of 310 of the children when they were 5 years old and 323 of the children at age 7. PBDE exposures were estimated based on measurements of maternal blood serum collected during the pregnancies and child blood serum at age 7 years.

In their analysis, the researchers adjusted for other factors that might affect neurodevelopment and be correlated with exposure, including maternal exposures to lead, PCBs, and organophosphate pesticides; they also incorporated assessments from parents and teachers of children’s attention, learning, and behavior. They found associations between maternal PBDE levels during pregnancy and evidence of deficits in children’s attention, fine motor coordination, and cognitive functioning at both ages. The children’s PBDE levels were associated with lower scores for full-scale IQ, particularly processing speed, verbal comprehension, and perceptual reasoning. The researchers also found that each 10-fold increase in the children’s total measured PBDE levels was associated with at least 4.5 times higher odds of the child being rated by teachers as at least moderately hyperactive and impulsive.

The researchers initially hypothesized that prenatal exposure to PBDEs would have a stronger influence on neurodevelopment than childhood exposures, so they considered it noteworthy that later exposures were associated with outcomes at age 7 independently of prenatal exposures. At 7 years of age, the children’s PBDE levels were three times higher than their mothers’ levels during pregnancy for the sum of the four most frequently detected congeners. The authors attribute this to the fact that the children had lived in California all their lives, whereas many of the mothers had come there recently, which points to the home environment as a major source of exposure.

